# Schiff bases containing a furoxan moiety as potential nitric oxide donors in plant tissues

**DOI:** 10.1371/journal.pone.0198121

**Published:** 2018-07-10

**Authors:** Emilian Georgescu, Anca Oancea, Florentina Georgescu, Alina Nicolescu, Elena Iulia Oprita, Lucian Vladulescu, Marius-Constantin Vladulescu, Florin Oancea, Sergiu Shova, Calin Deleanu

**Affiliations:** 1 Research Center Oltchim, RamnicuValcea, Romania; 2 National Institute of Research and Development for Biological Sciences, Bucharest, Romania; 3 Research Dept., Teso Spec S.R.L., Fundulea, Calarasi, Romania; 4 “PetruPoni” Institute of Macromolecular Chemistry, Romanian Academy, Aleea Grigore Ghica Voda, Iasi, Romania; 5 “C. D. Nenitescu” Centre of Organic Chemistry, Romanian Academy, Bucharest, Romania; 6 National Research & Development Institute for Chemistry & Petrochemistry – ICECHIM, Bucharest, Romania; 7 Institute of Chemistry, Academy of Sciences, Chisinau, Republic of Moldova; Boston University, UNITED STATES

## Abstract

Stable Schiff bases containing a furoxan moiety are synthesized as single regioisomers by the reaction of 3-methyl-2-oxy-furazan-4-carbaldehydewith various amino compounds at room temperature. The structures of synthesized compounds were fully characterized by multinuclear NMR spectroscopy and X-ray crystallography. The effect of synthesized Schiff bases containing a furoxan moiety on biological generation of reactive oxygen species and nitric oxide in plant tissues was investigated for the first time by fluorescence microscopy and the released NO identified as nitrite with Griess reagent. There is a good correlation between the biological generation of NO determined by fluorescence microscopy and with Griess reagent. Some of the synthesized compounds exhibited both nitric oxide and reactive oxygen species generation abilities and represent potential NO donors in plant tissues.

## Introduction

Nitric oxide (NO) is a signaling molecule common to animals and plants [1–2]. In plants, NO participates in important processes such as germination, flowering, stomatal closure [2–8], activates disease resistance to pathogen attacks and possibly acts as direct anti-microbial agent [9]. Plant defense responses to pathogen attacks is activated by a complex signal produced by the accumulation of reactive oxygen species (ROS) and NO [3–6]. These findings stand for eco-friendly means to control disease in plants. Many chemicals have been used as NO donors or even for biological generation of NO in animals and plants [10,11]. Sodium nitroprusside (SNP), *S*-nitrosoglutathione (GSNO), *S*-nitroso-*N*-acetylpenicillamine (SNAP) and so-called NONOates (spermidine or diethylamine-NONOate) are among the most used nitric oxide donors [12,13].

Searching for synthetic compounds acting as NO donors or as biological inducers of NO in plants is important not only for understanding the NO mechanism in plants but also for field applications. Our expertise on synthesis of new bioactive heterocyclic compounds [14–20] and interest in signaling compounds in plants [21,22] prompted us to obtain stable Schiff bases containing a furoxan moiety as possible NO donors in plants. Furoxan, 1,2,5-oxadiazole *N*-oxide, is an important scaffold of many compounds that show typical NO-donor properties in mammals, some furoxan derivatives being known as NO-donating pro-drugs [23–27]. Schiff bases are resourceful intermediates in several enzymatic reactions [17] as well as for the design of a large number of bioactive lead compounds [28,29]. Their biological properties include antibacterial [30,31], biocidal [32], antifungal [33], antiviral [33,34], and high antitumor activities [34–36].

Various synthetic methods towards furoxan derivatives such as the cyclization of *α*-nitro ketoximes [37,38], the dimerization of nitrile *N*-oxides [39], the reaction of alkenes with aqueous sodium nitrite in glacial acetic acid [40–42], and the reaction of styrene derivatives with nitrosonium tetrafluoroborate (NOBF_4_), usually leading to both furoxan regioisomers, were reported [43].

Herein, we present the synthesis of stable Schiff bases containing a furoxan moiety obtained as single regioisomer and their effects as nitric oxide donors in plant tissues.

## Materials and methods

### Analytical equipment

Melting points were measured on a Boëtius hot plate microscope and are uncorrected.

IR spectra were recorded on a Nicolet Impact 410 spectrometer, in KBr pellets.

The NMR spectra have been recorded on a Bruker Avance III 400 instrument operating at 400.1, 100.6 and 40.6 MHz for ^1^H, ^13^C, and ^15^N nuclei respectively. Samples were transferred in 5 mm Wilmad 507 NMR tubes and recorded with either a 5 mm multinuclear inverse detection z-gradient probe (^1^H spectra and all H-C/H-N 2D experiments) or with a 5 mm four nuclei direct detection z-gradient probe (^13^C spectra). Chemical shifts are reported in δ units (ppm) and were referenced to internal TMS for ^1^H chemical shifts, to the internal deuterated solvent for ^13^C chemical shifts (CDCl_3_ referenced at 77.0 ppm) and to liquid ammonia (0.0 ppm) using nitromethane (380.2 ppm) as external standard for ^15^N chemical shifts. Unambiguous 1D NMR signal assignments were made based on 2D NMR homo- and heterocorrelations.

High resolution MS spectra have been recorded on a Bruker Maxis II QTOF spectrometer with electrospray ionization (ESI) in the negative mode.

X-Ray crystallographic measurements were carried out with an Oxford-Diffraction XCALIBUR E CCD diffractometer equipped with graphite-monochromated Mo-Kα radiation. The crystal was kept at 200.00(10) K during data collection. The unit cell determination and data integration were carried out using the CrysAlis package of Oxford Diffraction [44]. Using Olex2 [45], the structure was solved with the ShelXT [46] structure solution program using Direct Methods and refined with the ShelXL [47] refinement package using Least Squares minimization. These data can be obtained free of charge via www.ccdc.cam.ac.uk/conts/retrieving.html (or from the Cambridge Crystallographic Data Centre, 12 Union Road, Cambridge CB2 1EZ, UK).

Crotonic aldehyde, *p*-toluenesulfonyl hydrazide, 2,4,6-trimethylbenzenesulfonyl hydrazide, 4-phenyl-3-thiosemicarbazide, 4-(4-methylphenyl)-3-thiosemicarbazide, *p*-toluic hydrazide were purchased from Sigma Aldrich and used further without purification.

Fluorescence measurements were recorded on a Zeiss AXIO—OBSERVER D1, equipped with a video digital camera AxioCamMRc using AxioVision Rel.4.6 software.

Spectrophotometric analysis were performed on the Elisa plate according the reported bioprotocol [48].

### Materials

Fluorescence indicator 2’,7’-dichlorodihydrofluorescein diacetate (H_2_DCFA) was purchased from Invitrogen Molecular Probes. Fluorescence indicator4-amino-5-methylamino-2’,7’-difluorofluorescein diacetate (DAF-FM DA), chitosan, TWEEN 20, a nonionic detergent, sulfanilamide and *N*-(1-naphthyl)ethylenediamine dihydrochloride for Griess reagent were purchased from Sigma Aldrich.

### General procedure for Schiff bases containing a furoxan moiety 3, 5 and 7

A solution of 25 mmol (3.5 g) of 3-methyl-2-oxy-furazan-4-carbaldehyde **1** in 30 mL methanol was added dropwise to a stirred solution containing 20 mmol of an amino derivative (**2**, **4** and **6** respectively) in 30 mL methanol. After four hours stirring at ambient temperature, the solvent was partly removed under vacuum. The formed solid was filtered off, washed with cold ethanol (5 mL) and recrystallized.

#### *p*-Toluenesulfonic acid (3-methyl-2-oxy-furazan-4-ylmethylene)hydrazide (3a)

White crystals, yield 86% (5.1 g), mp 163-165°C (from MeOH:H_2_O 1:1), IR (ν, cm^-1^): 3210, 1612, 1470, 1383, 1349, 1309, 1158, 1080, 1038. ^1^H NMR (400.1 MHz, DMSO-d6, δ (ppm)): 2.16 (3H, s, CH_3_-3), 2.40 (3H, s, CH_3_-12), 7.45 (2H, d, 8.0 Hz, H-11/13), 7.76 (2H, d, 8.0 Hz, H-10/14), 7.93 (1H, s, H-6), 12.35 (1H, s, NH-8). ^13^C NMR (100.6 MHz, DMSO-d6, δ (ppm)): 9.2 (CH_3_-3), 21.0 (CH_3_-12), 111.2 (C-3), 127.2 (CH-10/14), 129.9 (CH-11/13), 134.9 (CH-6), 135.4 (C-9), 144.1 (C-12), 153.7 (C-4). ^15^N NMR (40.6 MHz, DMSO-d6, δ (ppm)): 175.4 (NH-8), 340.2 (N-7), 357.8 (N-2), 373.1 (N-5). HRMS-ESI (*m*/*z*): [M-H]^-^for C_11_H_11_N_4_O_4_S, calcd. 295.0501, found 295.0518.

#### 2,4,6-trimethylbenzenesulfonic acid (3-methyl-2-oxy-furazan-4-ylmethylene)hydrazide (3b)

Pale yellow solid, yield 74% (4.8 g), mp 177-179°C (from MeOH). IR (ν, cm^-1^): 3199, 2979, 2936, 1608, 1467, 1377, 1330, 1301, 1162, 1098, 1029. ^1^H NMR (400.1 MHz, DMSO-d6, δ (ppm)): 2.04 (3H, s, CH_3_-3), 2.28 (3H, s, CH_3_-12), 2.60 (6H, s, CH_3_-10/14), 7.10 (2H, s, H-11/13), 7.94 (1H, s, H-6), 12.53 (1H, s, NH-8). ^13^C NMR (100.6 MHz, DMSO-d6, δ (ppm)): 8.7 (CH_3_-3), 20.4 (CH_3_-12), 22.5 (CH_3_-10/14), 111.1 (C-3), 131.7 (CH-11/13), 132.4 (C-9), 133.3 (CH-6), 139.3 (C-10/14), 142.9 (C-12), 153.3 (C-4). ^15^N NMR (40.6 MHz, DMSO-d6, δ (ppm)): 177.5 (NH-8), 338.5 (N-7), 357.6 (N-2), 371.6 (N-5). X-Ray:C_13_H_16_N_4_O_4_S, (*M* = 324.36 g/mol): monoclinic, space group P2_1_/n (no. 14), *a* = 7.8827(6) Å, *b* = 18.4644(14) Å, *c* = 10.6000(8) Å, *β* = 110.634(9), *V* = 1443.9(2) Å^3^, *Z* = 4, *T* = 200.00(10) K, μ(MoKα) = 0.241 mm^-1^, *Dcalc* = 1.469 g/cm^3^, 5588 reflections measured (4.412 ≤ 2Θ ≤ 50.046), 2552 unique (*R*_int_ = 0.0303, R_sigma_ = 0.0558) which were used in all calculations. The final *R*_1_ was 0.0556 (I ≥ 2σ(I)) and *wR*_2_ was 0.1466 (all data).CCDC – 1556711. HRMS-ESI (*m*/*z*): [M-H]^-^ for C_13_H_15_N_4_O_4_S, calcd.323.0814, found 323.0846.

#### 4-methyl-benzoic acid (3-methyl-2-oxy-furazan-4-ylmethylene)hydrazide (5)

White solid, yield 71% (3.7 g), mp 212-214°C (from MeOH), IR (ν, cm^-1^): 3422, 3220, 3032, 1638, 1610, 1570, 1490, 1460, 1381, 1331, 1310, 1283, 1186, 1147, 1036. ^1^H NMR (400.1 MHz, DMSO-d6, δ (ppm)): 2.40 (6H, bs, CH_3_-3 and CH_3_-13), 7.38 (2H, d, 7.1 Hz, H-12/14), 7.85 (2H, d, 7.1 Hz, H-11/15), 8.49 (1H, s, H-6), 12.33 (1H, s, NH-8). ^13^C NMR (100.6 MHz, DMSO-d6, δ (ppm)): 9.1 (CH_3_-3), 21.1 (CH_3_-13), 111.7 (C-3), 127.8 (CH-11/15), 129.2 (CH-12/14), 129.6 (C-10), 135.8 (CH-6), 142.6 (C-13), 154.1 (C-4), 163.2 (CO-9). ^15^N NMR (40.6 MHz, DMSO-d6, δ (ppm)): 174.0 (NH-8). HRMS-ESI (*m*/*z*): [M-H]^-^ for C_12_H_11_N_4_O_3_, calcd. 259.0831, found 259.0851.

#### 1-(3-methyl-2-oxy-furazan-4-ylmethylene)-4-phenyl-3-thiosemicarbazone (7a)

Beige solid, yield 69% (3.83 g), mp 184-186°C (from CHCl_3_/MeOH), IR (ν, cm^-1^): 3324, 3128, 2983, 1609, 1518, 1491, 1462, 1383, 1306, 1251, 1169, 1110, 1033. ^1^H NMR (400.1 MHz, DMSO-d6, δ (ppm)): 2.44 (3H, s, CH_3_-3), 7.24 (1H, t, 7.6 Hz, H-14), 7.40 (2H, t, 7.6 Hz, H-13/15), 7.59 (2H, d, 7.6 Hz, H-12/16), 8.24 (1H, s, H-6), 9.87 (1H, s, NH-10), 12.32 (1H, s, NH-8). ^13^C NMR (100.6 MHz, DMSO-d6, δ (ppm)): 9.2 (CH_3_-3), 111.6 (C-3), 125.1 (CH-12/16), 125.6 (CH-14), 128.3 (CH-13/15), 131.4 (CH-6), 138.7 (C-11), 153.9 (C-4), 176.5 (CS-9). ^15^N NMR (40.6 MHz, DMSO-d6, δ (ppm)): 129.1 (NH-10), 177.4 (NH-8), 334.1 (N-7), 357.6 (N-2), 372.3 (N-5). HRMS-ESI (*m*/*z*): [M-H]^-^ for C_11_H_10_N_5_O_2_S, calcd. 276.0555, found 276.0537.

#### 1-(3-methyl-2-oxy-furazan-4-ylmethylene)-4-(4-methylphenyl)-3-thiosemicarbazone (7b)

Yellow solid, yield 63% (3.67 g), mp 188-190°C (MeOH/Et_2_O), IR (ν, cm^-1^): 3412, 3350, 3127, 2966, 1612, 1540, 1517, 1458, 1379, 1304, 1259, 1205, 1170, 1120, 1024. ^1^H NMR (400.1 MHz, DMSO-d6, δ (ppm)): 2.31 (3H, s, CH_3_-14), 2.43 (3H, s, CH_3_-3), 7.19 (2H, d, 8.2 Hz, H-13/15), 7.45 (2H, d, 8.2 Hz, H-12/16), 8.23 (1H, s, H-6), 9.78 (1H, s, NH-10), 12.26 (1H, s, NH-8). ^13^C NMR (100.6 MHz, DMSO-d6, δ (ppm)): 9.2 (CH_3_-3), 20.5 (CH_3_-14), 111.6 (C-3), 125.1 (CH-12/16), 128.7 (CH-13/15), 131.2 (CH-6), 134.9 (C-14), 136.1 (C-11), 153.9 (C-4), 176.5 (CS-9). ^15^N NMR (40.6 MHz, DMSO-d6, δ (ppm)): 128.8 (NH-10), 177.4 (NH-8), 334.4 (N-7), 357.6 (N-2), 372.6 (N-5). HRMS-ESI (*m*/*z*): [M-H]^-^ for C_12_H_12_N_5_O_2_S, calcd. 290.0712, found 290.0722.

### Histochemical analysis

#### Plant growth conditions and treatment

In our experiments we used *Arabidopsis thaliana* plants, cultivated in laboratory in Arasystem [49]. *Arabidopsis thaliana* wild type seeds (provided by *Lehke Seeds Texas*, *USA*) have been seeded in sterilized soil and cultivated for six weeks in a special growth room, at 21-23°C, 70% humidity, light intensity 150 μmol/m^2^ and a photoperiode of 14/10. Each synthesized compound (0.5 mg, and 2.5 mg respectively) dissolved in ethanol was mixed with 0.25 g Tween 20 and demineralized water to prepare 50 mL of each test suspension/solution. The inductor suspensions were kept in spraying glass bottle, in the dark, at room temperature. The *Arabidopsis* leaves were sprayed with the inductor suspensions (at a rate of 1 mL/plant) andcollected after 24 hours. The leaves were washed with distilled water for histochemical analysis of ROS and NO by fluorescence microscopy, or worked-up according to the reported protocol [48], in order to determine NO releasing potential of new synthesized Schiff bases bearing a furoxan moiety with Griess reagent. As positive control in histochemical analysis by fluorescent microscopy we used plant treated with chitosan solution, 10 μg/mL, and 50 μg/mL respectively, in 0.5% acetic acid solution, buffered to pH 5.6 with NaOH 1 M.

#### ROS and NO visualization by fluorescence microscopy

Intracellular ROS was visualized using 2’,7’-dichlorodihydrofluorescein diacetate (H_2_DCFA) as fluorescent indicator. The collected *Arabidopsis* leaves were washed with distilled water and incubated with 2.5 μMH_2_DCFA solution (10 mMin DMSO), for 30 min, in the dark, at room temperature. Then the leave fragments were washed twice with distilled water and the H_2_DCFA – mediated fluorescence was detected (emission/excitation: 488/525 nm). As negative controls, *Arabidopsis* leaves untreated with inductor suspensions have been used. Intracellular NO was visualized using 4-amino-5-methylamino-2’,7’-difluorofluorescein diacetate (DAF-FM DA)as fluorescent indicator. The collected *Arabidopsis* leaves were washed with distilled water and incubated with 10 μM DAF-FM diacetate (5mM in DMSO),for 15 min, in the dark, at room temperature. Then the leave fragments were washed twice with phosphate buffer saline (PBS) at pH 7.4 and the fluorescence of the reaction product of DAF-FM DA with NO was captured (emission/excitation: 488/525 nm). As negative controls, *Arabidopsis* leaves untreated with inductor suspensions have been used.

The NO specific dye, 4-amino-5-methylamino-2’,7’-difluorofluorescein (DAF-FM), reacts with N_2_O_3_, generated by NO oxidation, and form a DAF-FM benzotriazole derivative which exhibits a green fluorescence. However, this dye is not cell permeant and its fluorescent derivatives are an indication of ROS (and NO) formation outside of the plant cells, on tissue level. The 4-amino-5-methylamino-2’,7’-difluorofluorescein diacetate (DAF-FM DA) is a cell permeable dye. This dye is converted by cytosolic esterase to DAF-FM, which produces the benzotriazole fluorescent derivative inside the cell. The generation of ROS is a biological effect in plants, mainly due to the released NO, which are redox gasotransmitters. The reactive species formed in plants are NO and, most probably, on physiological conditions, peroxynitrite (due to NO reaction with ROS).

#### Determination of nitrite concentration in *Aradidopsis thaliana* leaves with Griess reagent

50 μl of sulfanilamide 1% (w/v) solution in 5% (v/v) phosphoric acid and 50 μl of *N*-(1-naphthyl)ethylenediamine dihydrochloride 0.1% (w/v) solution were added to 50 μl *Arabidopsis* leaves extract supernatant. The leaves, treated with the same amounts of synthesized Schiff bases suspensions, collected after 24 hours, and controls washed with distilled water, were powdered with nitrogen liquid into a mortar. 100 mg of leaves powder was extracted for 30 min in 300 μl of 100 mM phosphate buffer, pH 7.4. The extract was centrifuged for 15 min at 10,000 x g and 4°C. The resulted supernatant was used for indirect NO determination with sulfanilamide and *N*-(1-naphthyl)ethylenediamine dihydrochloride solutions, after incubation for 5-10 min at room temperature protected from light. The color appeared immediately as the Griess reagent is formed. The absorbance was directly measured in a plate reader with a filter between 520 nm and 550 nm. A nitrite standard curve was used to calculate the nitrite concentration in the samples and expressed as μM of nitrite anion. The detection of nitrite concentration in *Aradidopsis thaliana* leaves was performed according to the reported bioprotocol [48].

## Results and discussion

### Synthesis of Schiff bases containing a furoxan moiety

**Fig 1:**
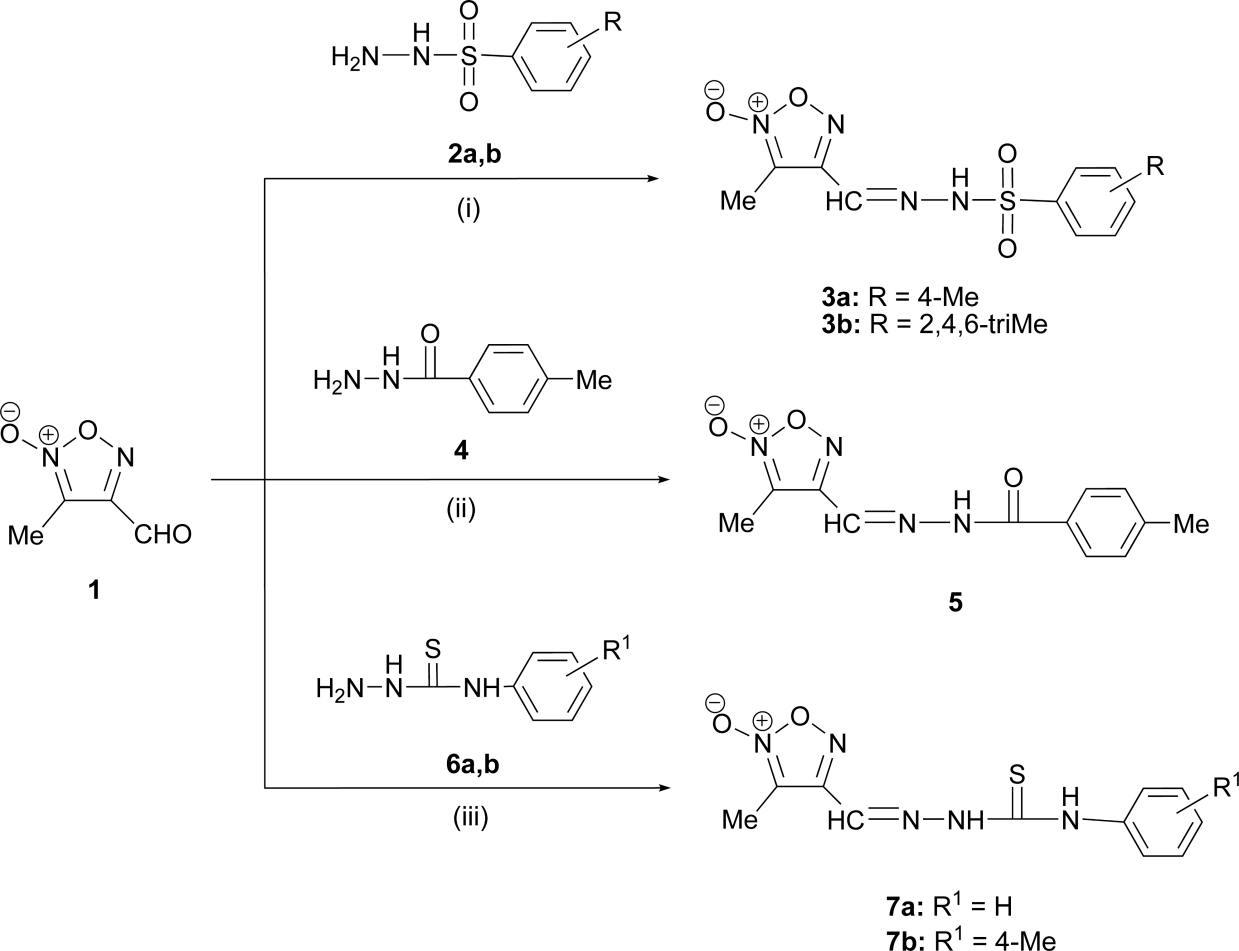
Synthesis of Schiff bases containing a furoxan ring.

Stable Schiff bases containing a furoxan moiety were synthesized in order to explore their chemical and biological properties. The synthetic procedure is based on the reactions of the 3-methyl-2-oxy-furazan-4-carbaldehyde with various amino compounds capable to produce stable Schiff bases bearing a furoxan ring. The intermediate 3-methyl-2-oxy-furazan-4-carbaldehyde (**1**), already described in literature [40], was easily obtained as single isomer from crotonic aldehyde and sodium nitrite in glacial acetic acid at room temperature. Therefore, by treating furoxan carbaldehyde **1** with phenylsulfonyl hydrazide derivatives **2a,b** the corresponding phenylsulfonylhydrazones containing a furoxan moiety **3a,b** were obtained (Fig 1i). Starting from the furoxan carbaldehyde **1** and *p*-toluic hydrazide **4** the corresponding Schiff base **5** bearing the furoxan ring was prepared (Fig 1ii). In the same way, the reaction of furoxan carbaldehyde **1** with thiosemicarbazides **6** led to the corresponding thio-semicarbazones **7a,b** carrying a furoxan moiety (Fig 1iii). All reactions took place easily at room temperature and yields are in the range 63-86%.

The structures of all Schiff bases containing the furoxan moiety were assigned on the basis of chemical and spectral analysis (IR, ^1^H, ^13^C and ^15^N NMR spectra). NMR data clearly indicated the presence of only one regioisomer bearing the external oxygen atom on the nitrogen in position 2 of the furoxan ring. The 2- *versus*5- *N*-oxidation of the furoxan ring in all compounds (**3**, **5**, **7**) is supported by similar chemical shifts for C3 and C4 in the ^13^C-NMR spectra. Assigning the site of *N*-oxidation in various natural or biological active compounds is important both for structural and mechanistic purposes related to metabolisation of these compounds. We have also previously investigated the influence of *N*-oxidation on ^15^N- and ^13^C-NMR spectra for series of octahydroacridines [50,51]. The shifts induced by *N*-oxidation to C-alpha (C3 in furoxan derivatives) and C-beta (C4 in furoxan derivatives) is consistent with our previous studies [51], and with early data on simple furoxan derivatives [52]. The *N*-oxidation induces a significant shielding of the C-alpha and a slight deshielding of the C-beta in the ^13^C-NMR spectra.

**Fig 2:**
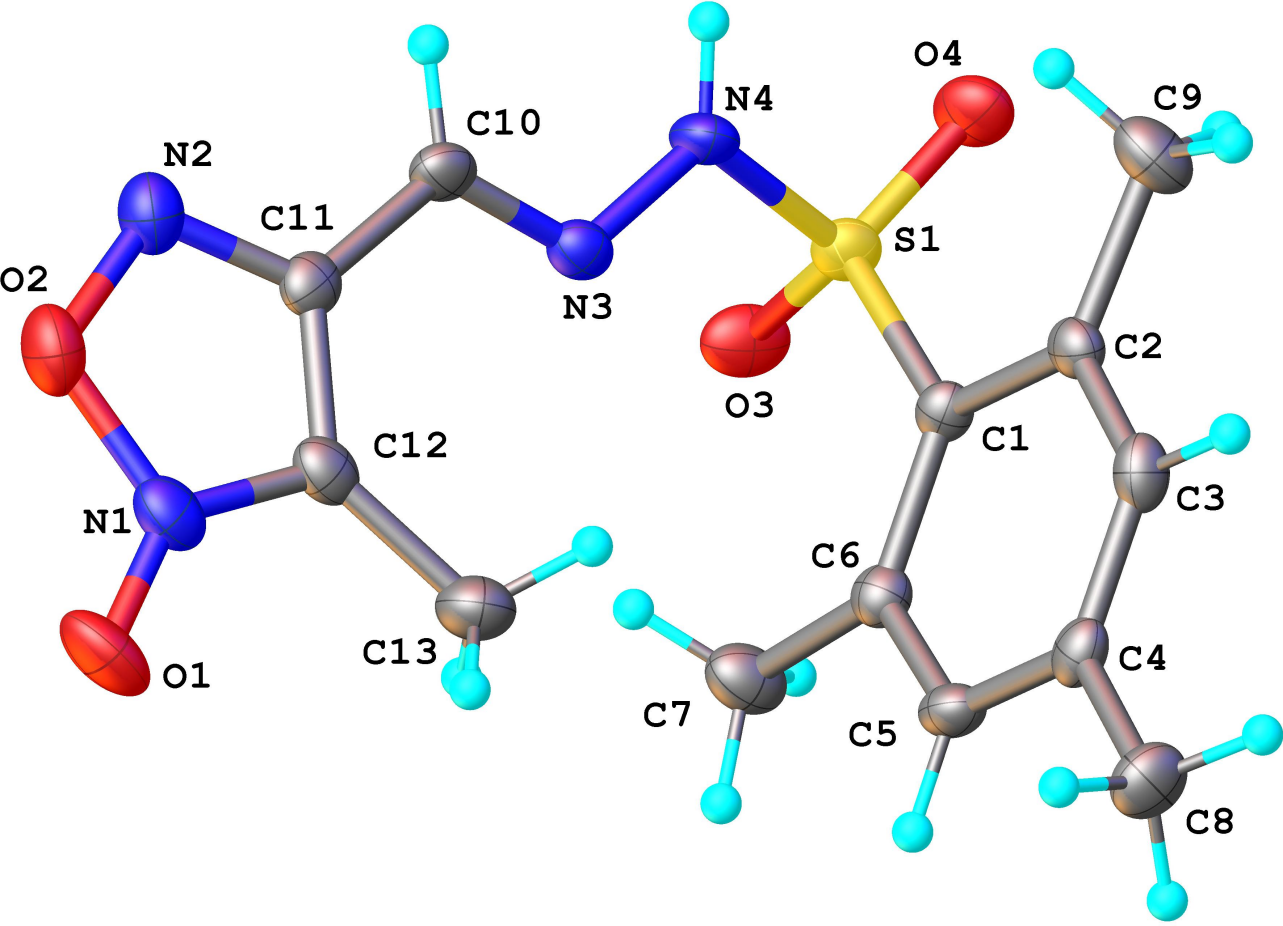
X-ray molecular structure of compound 3b. Thermal ellipsoids are drawn at 50% probability level.

The *N*-oxidation in position 2 of the furoxan ring in the case of derivative **3b** has been also proven by X-ray crystallography (Fig 2).

#### Biological activity

Furoxan derivatives have been of considerable interest to chemists for years but they received relatively little attention from biologists despite their NO-releasing capacities. It is for the first time when Schiff bases containing a furoxan moiety were used as NO donors in plant experiments. We investigated the effect of synthesized compounds on ROS (O_2_^−^, OH• and H_2_O_2_) and NO generation in plant tissues. Bio-molecules are rapidly damaged by reactive oxygen species produced under a pathogen attack or in abiotic stress conditions. It is known that both ROS and NO together are required to induce the activation of defense-related enzymes in plants [3]. The protection of plant cells at the sites of ROS and NO generation is ensured by both oxygen radical detoxifying enzymes and non-enzymatic antioxidants contained in plant cells [53,54]. The measurement of the ROS and NO levels in plant tissues is difficult due to very short physiological half-life and high reactivity of these radicals [12,55].

Both ROS and NO were detected by the fluorescence microscopy on *Arabidopsisthaliana*, a popular model organism for understanding themolecular biologyof many plant traits. Specific fluorescence indicators that are helpful to exactly define the sites of NO and ROS production were used. The presence of ROS and NO in Schiff bases bearing a furoxan moiety-treated *Arabidopsis* leaves was compared to untreated *Arabidopsis* leaves as a negative control. Chitosan, a fungal elicitor with known effect as NO and ROS inductor on *Arabidopsis* [56], was used as positive control at the same concentrations.

ROS induction was detected on *Arabidopsis* leaves treated with suspension of each synthesized Schiff bases containing a furoxan moiety at the concentration of 10 μg/mL, and 50 μg/mL respectively, in the presence of the specific fluorescence indicator 2’,7’-dichlorodihydrofluorescein diacetate (H_2_DCFA) [57]. Fluorescence microscopy images revealed the presence of ROS in *Arabidopsis* leaves treated with all Schiff bases having a furoxan moiety, at both concentrations, especially at higher concentration of compounds (50 μg/mL). Efficacy of Schiff bases bearing a furoxan moiety (**3a**,**b**, **5**, **7a** and **7b**) on ROS generation pursues the series: **7a**>**3b**≥**7b**>**5**>**3a**.

NO donor properties of the synthesized Schiff bases bearing a furoxan moiety were determined on *Arabidopsis* leaves infiltrated with suspension of each synthesized compound at the same concentrations (10 μg/mL and 50 μg/mL respectively) in the presence of a specific and sensitive fluorescence indicator, 4-amino-5-methylamino-2’,7’-difluorofluorescein diacetate (DAF-FM DA) [57–60] and the DAF-FM DA- mediated fluorescence was measured. Strong fluorescence densities were observed at higher concentration (50 μg/mL) of Schiff bases bearing a furoxan moiety with their NO donor efficacy, following the series: **3b**>**7b**>**5**>**7a**>**3a**.

In order to assess the NO releasing potential in *Arabidopsis* leaves we used Griess reagent for indirect determination of NO through its oxidized nitrite form [48]. All *Arabidopsis* leaves treated with the same amounts of synthesized Schiff bases bearing a furoxan moiety were incubated at room temperature for 5-10 min. with Griess reagent, protected from light, and the absorbance was immediately measured in a plate reader with a filter between 520 nm and 550 nm. A nitrite standard curve was used to calculate the nitrite concentration in the samples and expressed as μM of nitrite anion (Table 1).

**Table 1:** NO releasing potential of synthesized Schiff bases bearing a furoxan moiety.

Comp. no.	Concentration (μg/mL)	NO releasing (μM NO_2_^-^)
Negative control	-	0.120
****3a****	50	0.500
****3b****	50	10.759
****5****	50	1.172
****7a****	50	2.147
****7b****	50	5.082

The fluorescence data on NO releasing capacity of these compounds in *Arabidopsisthaliana* leaves correlate with spectrophotometric data obtained by indirectly assessing NO as nitrite anion with Griess reagent.

All data suggest that some of the synthesized Schiff bases containing a furoxan moiety are involved in ROS and NO production in *Arabidopsis* treated leaves. Among these, compounds **3b** and **7b** proved to be really active and are further tested in field trials.

Considering the long-lasting effect, most probably NO release is not only a result of furoxanes decomposition, being rather specific to plant tissue. The NO released from furoxanes could accumulate as S-nitrothiols / S-nitroso-glutathione NO-reservoirs and then slowly released and detected by fluorescence microscopy or with Griess reagent. Thus, plant cells could have a physiological reaction to the Schiff base containing a furoxan moiety. Further research is in progress to assess the mechanism of NO generation into plant tissues by these compounds.

## Conclusions

Several Schiff bases containing a furoxan ring have been synthesized as single regioisomers starting from 3-methyl-2-oxy-furazan-4-carbaldehyde and various amino compounds capable to produce stable Schiff bases, in order to identify their chemical and biological properties. We detected for the first time ROS and NO releasing capacities in plant tissues using specific fluorescence indicators, and assessed the NO releasing potential of Schiff bases containing a furoxan ring treated *Arabidopisis thaliana* leaves. There is a good correlation between fluorescence data and indirect determination of NO biological releasing potential data in plant tissues. The results indicate that some of these compounds represent potential NO donors in plant tissues.

## Supporting information

S1 File Crystallographic information file (CIF) for the compound 3b. (CIF)

S2 File Fluorescence Microscopy and NMR information file. (DOC)
