# Supplementary material for: Schiff bases containing a furoxan moiety as potential nitric oxide donors in plant tissues
Source: PLoS One. 2018 Jul 10;13(7):e0198121. doi: 10.1371/journal.pone.0198121 (PMC6038987; doi:10.1371/journal.pone.0198121)
Supplement: S2 File — (DOC) [file pone.0198121.s002.doc]

**Supporting Information**

Schiff bases containing a furoxan moiety as potential nitric oxide donors in plant tissues

**Emilian Georgescu,^a^ Anca Oancea,^b^ Florentina Georgescu,^c^ Alina Nicolescu,^d.e^ Elena Iulia Oprita,^b^ Lucian Vladulescu,^c^ Marius-Constantin Vladulescu,^c^ Florin Oancea,^f^ Sergiu Shova^d,g^ and Calin Deleanu*^d,e^**

*^a^ Research Center Oltchim, Str. Uzinei 1, RO-240050, Ramnicu Valcea, Romania.*

*^b^ National Institute of Research and Development for Biological Sciences, Spl. Independentei 296, RO-060031 Bucharest, Romania.*

*^c^ Research Dept., Teso Spec S. R. L., Str. Muncii 53, RO-915200 Fundulea, Calarasi, Romania.*

*^d^ “Petru Poni” Institute of Macromolecular Chemistry, Romanian Academy, Aleea Grigore Ghica Voda 41-A, RO-700487 Iasi, Romania.*

*^e^ “C. D. Nenitescu” Centre of Organic Chemistry, Romanian Academy, Spl. Independentei 202-B, RO-060023 Bucharest, Romania.*

*^f^ National Research & Development Institute for Chemistry & Petrochemistry – ICECHIM, Spl. Independentei 202, RO-060021 Bucharest, Romania.*

*^g^ Institute of Chemistry, Academy of Sciences, Str. Academiei 3, MD-2028, Chisinau, Republic of Moldova.*

**Fluorescence Microscopy**

ROS induction was detected on *Arabidopsis* leaves treated with suspension of each synthesized Schiff bases containing a furoxan moiety at the concentration of 10 μg/mL, and 50 μg/mL respectively, in the presence of the specific fluorescence indicator 2’,7’-dichlorodihydrofluorescein diacetate (h_2_dcfa). Fluorescence microscopy images revealed the presence of ROS in *Arabidopsis* leaves treated with all Schiff bases having a furoxan moiety, at both concentrations, especially at higher concentration of compounds (50 μg/mL). Efficacy of Schiff bases bearing a furoxan moiety (**3a,b**, **5, 7a** and **7b**) on ROS generation pursues the series: **7a>3b≥7b>5>3a** (Figure S1).

NO donor properties of the synthesized Schiff bases bearing a furoxan moiety were determined on *Arabidopsis* leaves infiltrated with suspension of each synthesized compound at the same concentrations (10 μg/mL and 50 μg/mL respectively) in the presence of a specific and very sensitive fluorescence indicator, 4-amino-5-methylamino-2’,7’-difluorofluorescein diacetate (DAF-FM DA) and the DAF-FM DA - mediated fluorescence was measured. Strong fluorescence densities were observed at higher concentration (50 μg/mL) of Schiff bases bearing a furoxan moiety. Schiff bases bearing a furoxan moiety, listed in the increasing order of its NO donor efficacy, follows the series: **3b>7b>5>7a>3a** (Figure S2).


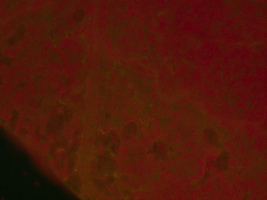


**NC**

**PC**


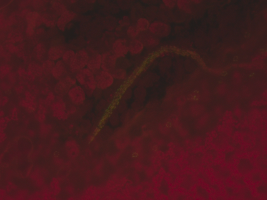

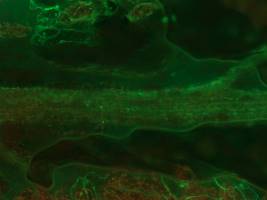


**3a**a

**3b**a


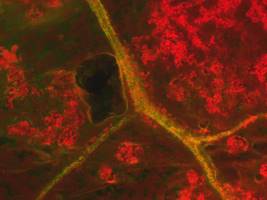

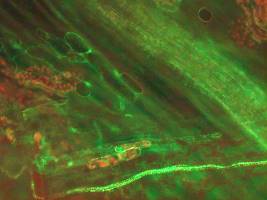


**5**

**7a**


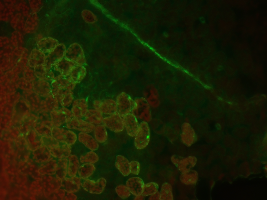


**7b**

**Figure S1** ROS generation in *Arabidopsis* leaves after 24 hours from treatment with donor compound suspensions (suspension concentration: 50 μg/mL; NC = negative control). Scale bar = 20 µm.


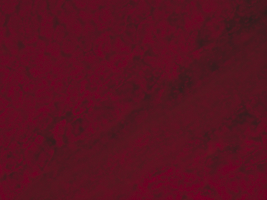

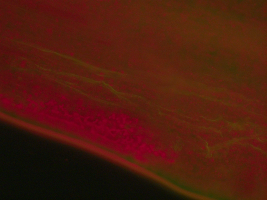


**NC**

**PC**


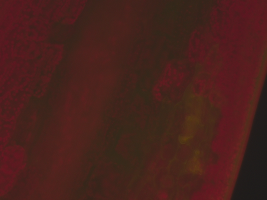

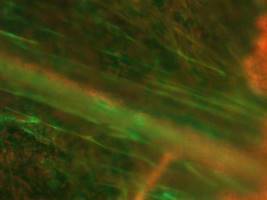


**3a**

**3b**


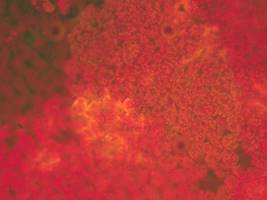

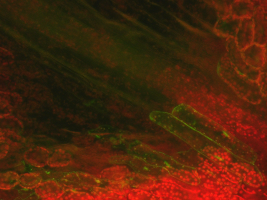


**5**

**7a**


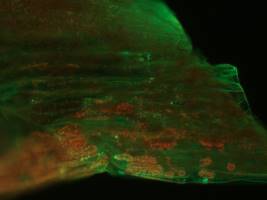


**7b**

**Figure S2** NO generation in *Arabidopsis* leaves after 24 hours from treatment with donor compound suspensions (suspension concentration: 50 μg/mL; NC = negative control; PC = positive control). Scale bar = 20µm.

**^13^C and H^1^ NMR spectra**


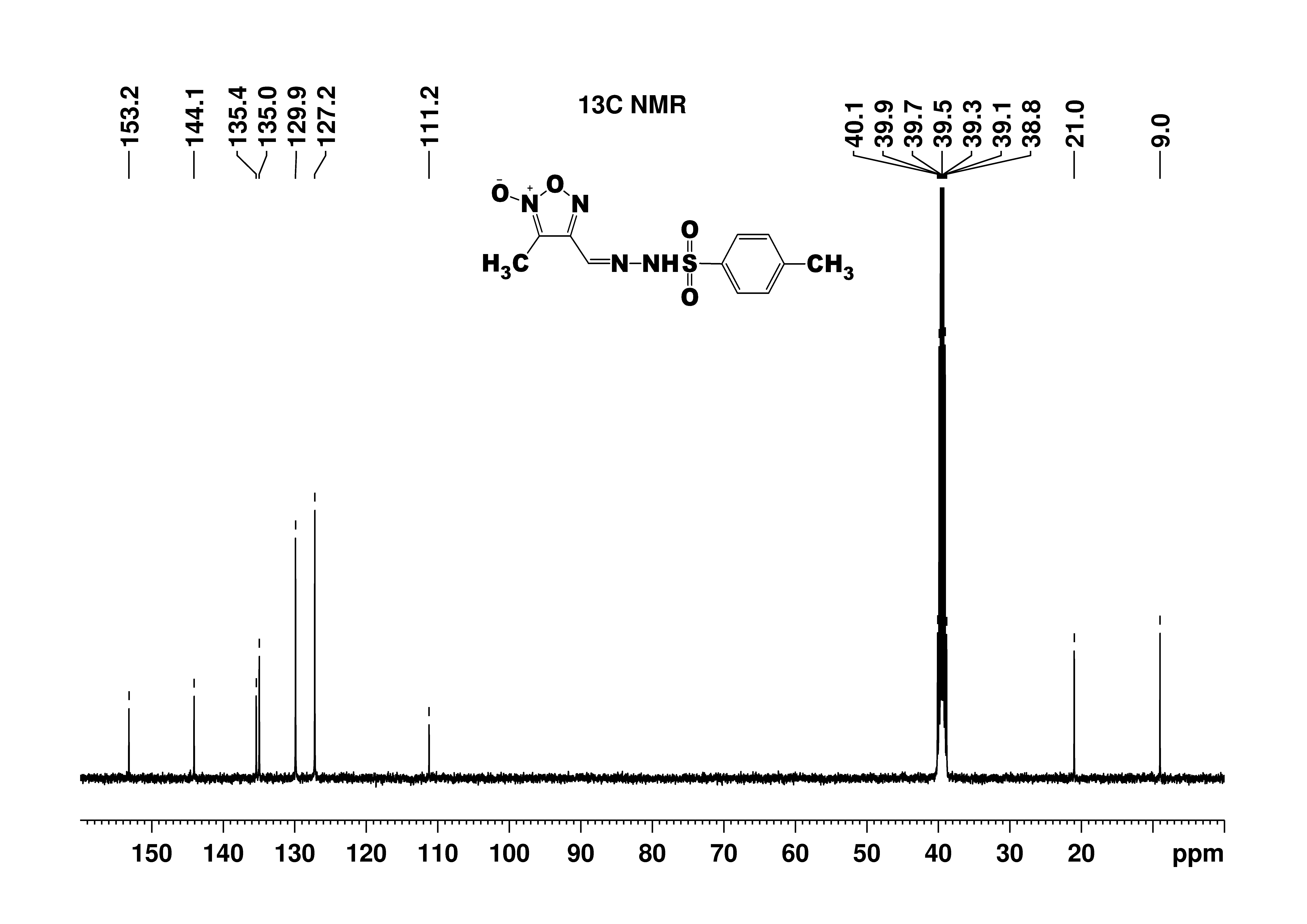


**Figure S3** ^13^C-NMR for Compound **3a** in DMSO-d6.


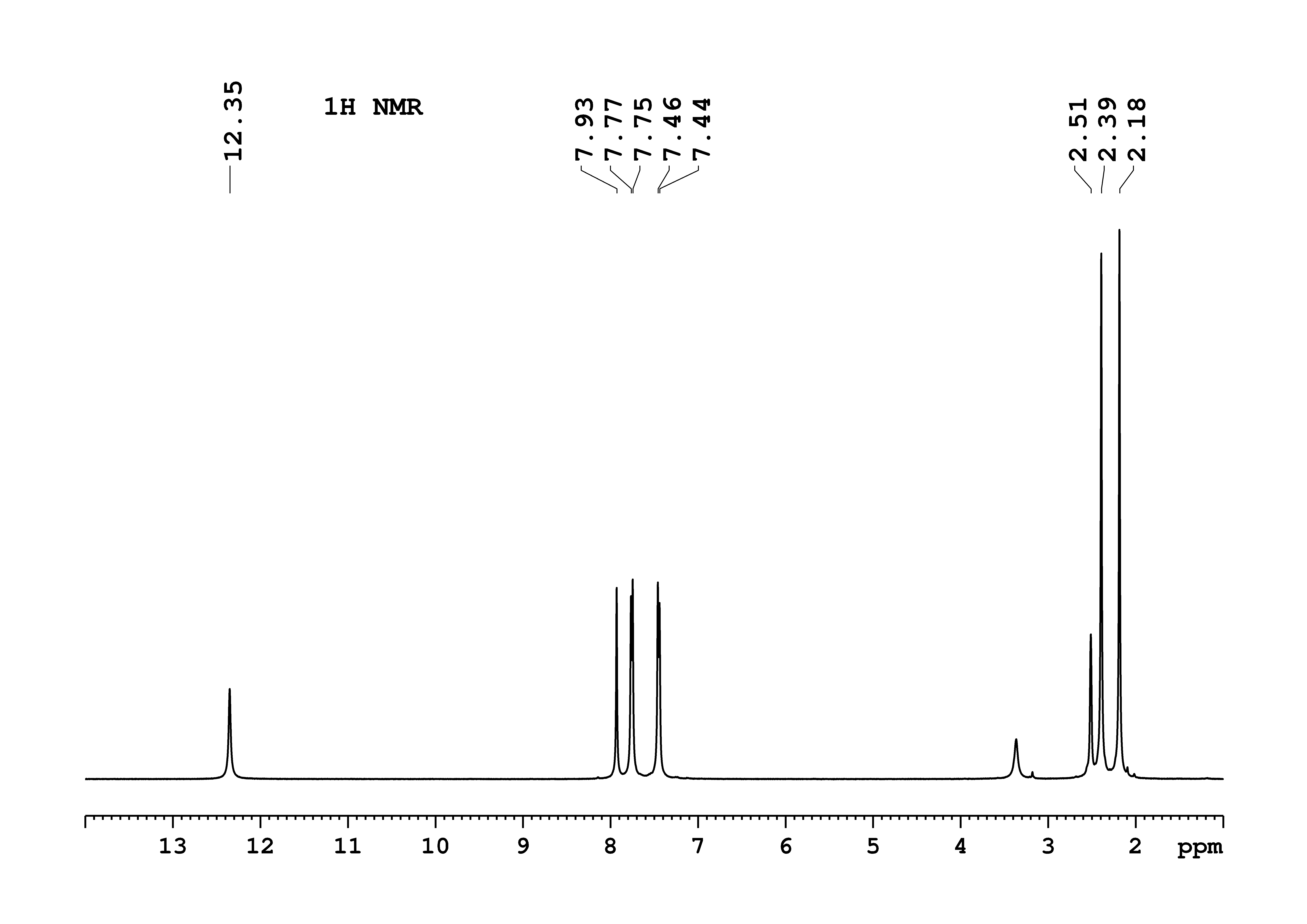


**Figure S4** ^1^H-NMR for Compound **3a** in DMSO-d6.

**Figure S5** ^15^N-NMR for Compound **3a** in DMSO-d6.


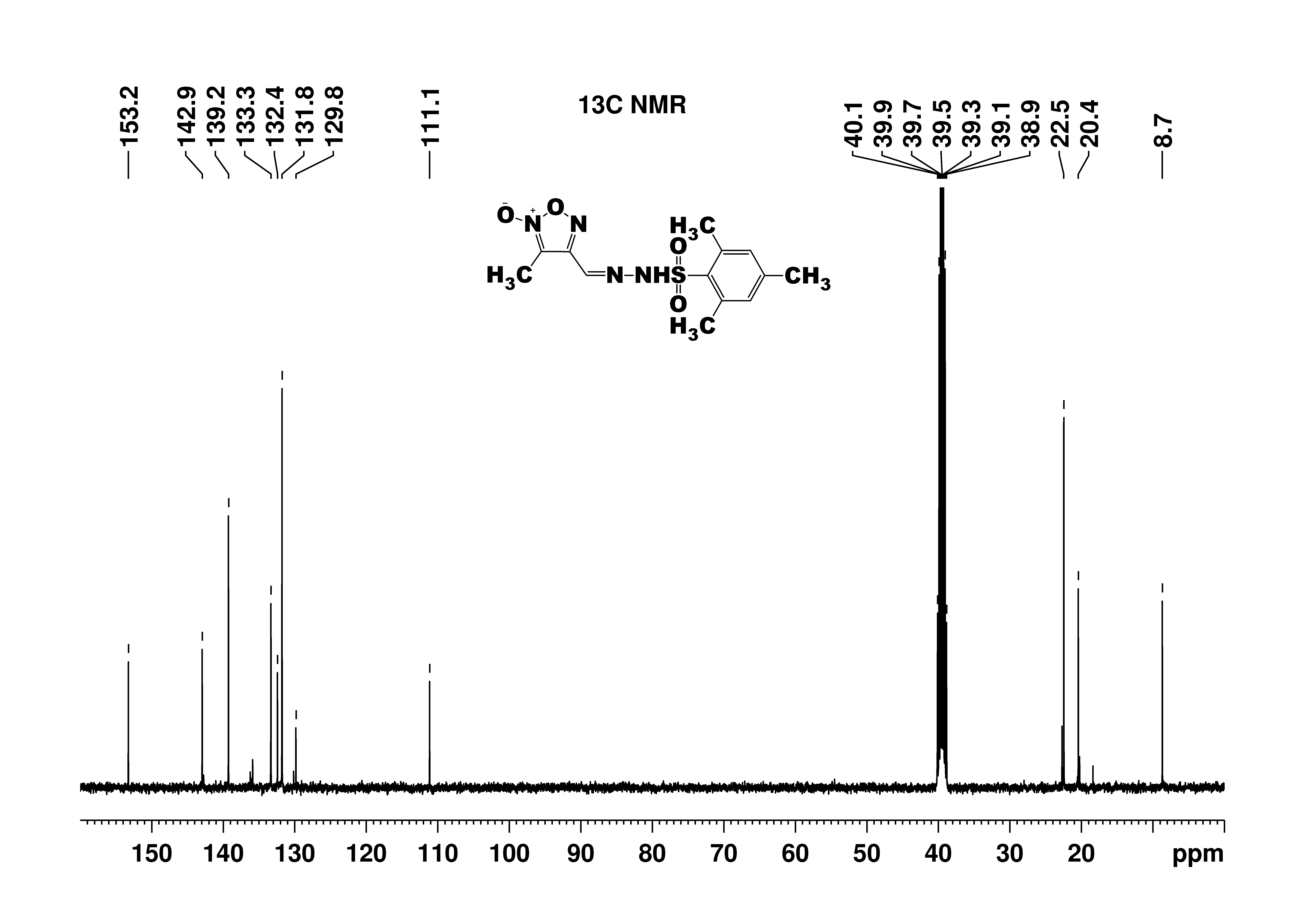


**Figure S6** ^13^C-NMR for Compound **3b** in DMSO-d6.


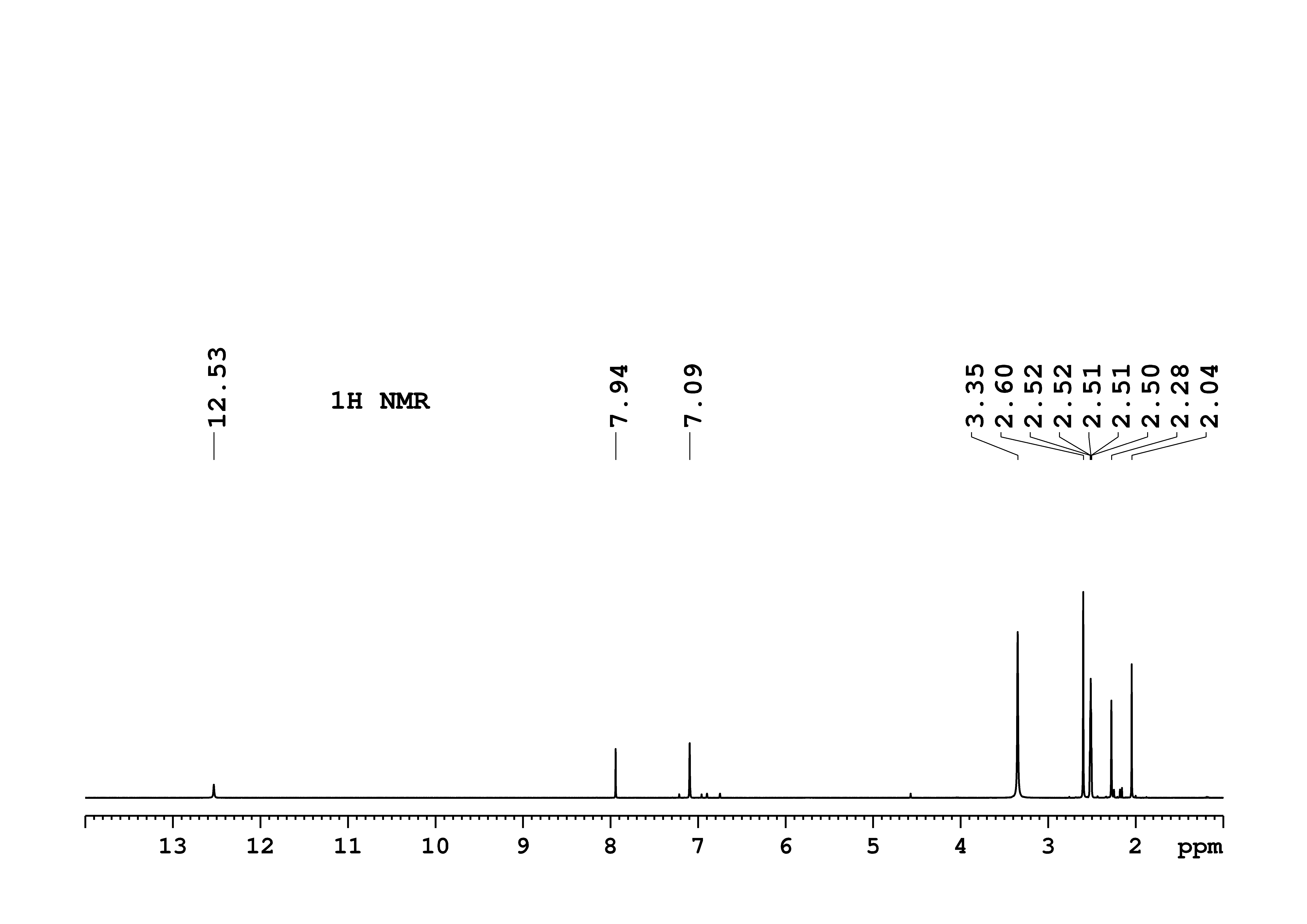


**Figure S7** ^1^H-NMR for Compound **3b** in DMSO-d6.


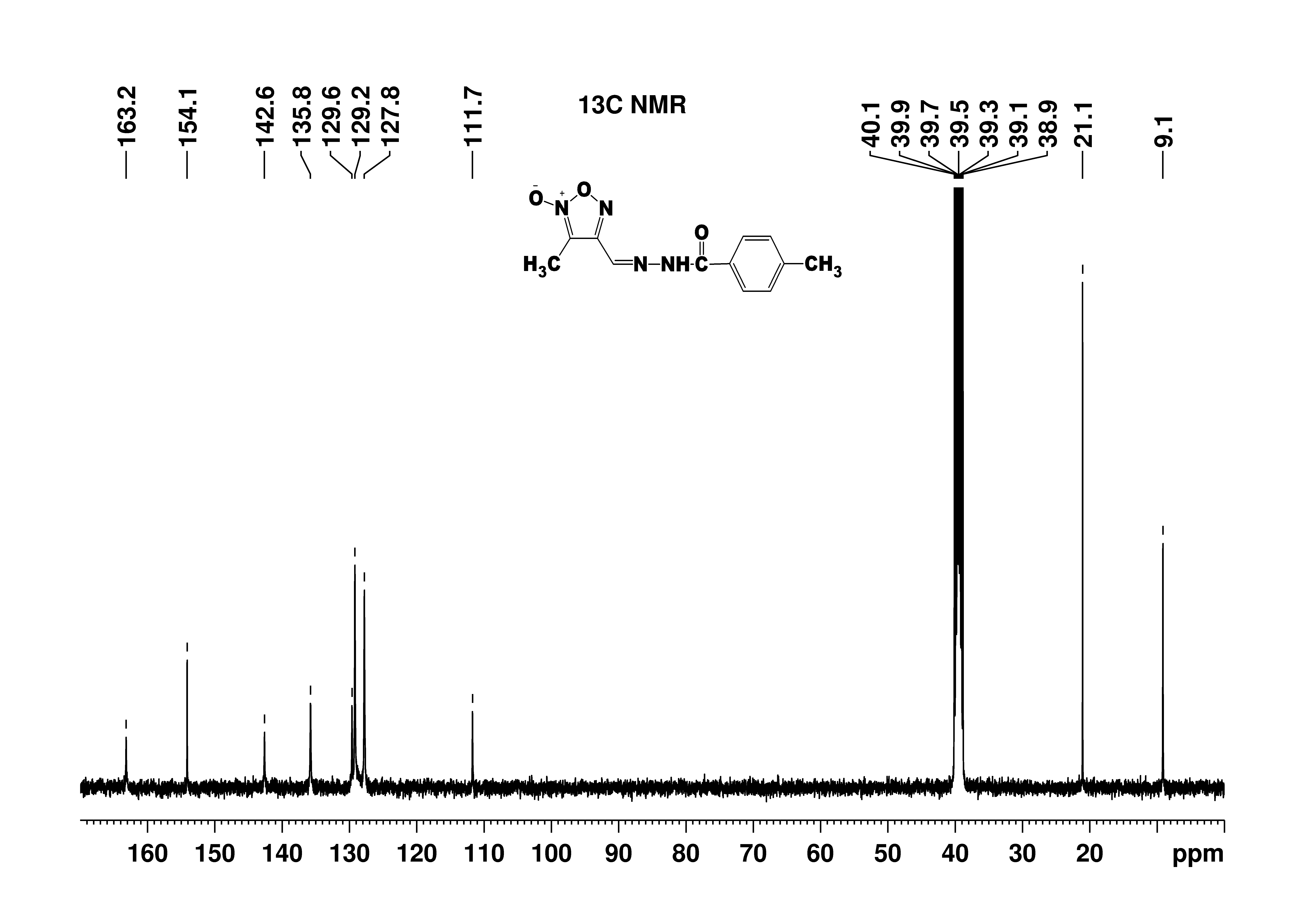


**Figure S8** ^13^C-NMR for Compound **5** in DMSO-d6.

**Figure S9** ^15^N-NMR for Compound **5** in DMSO-d6.


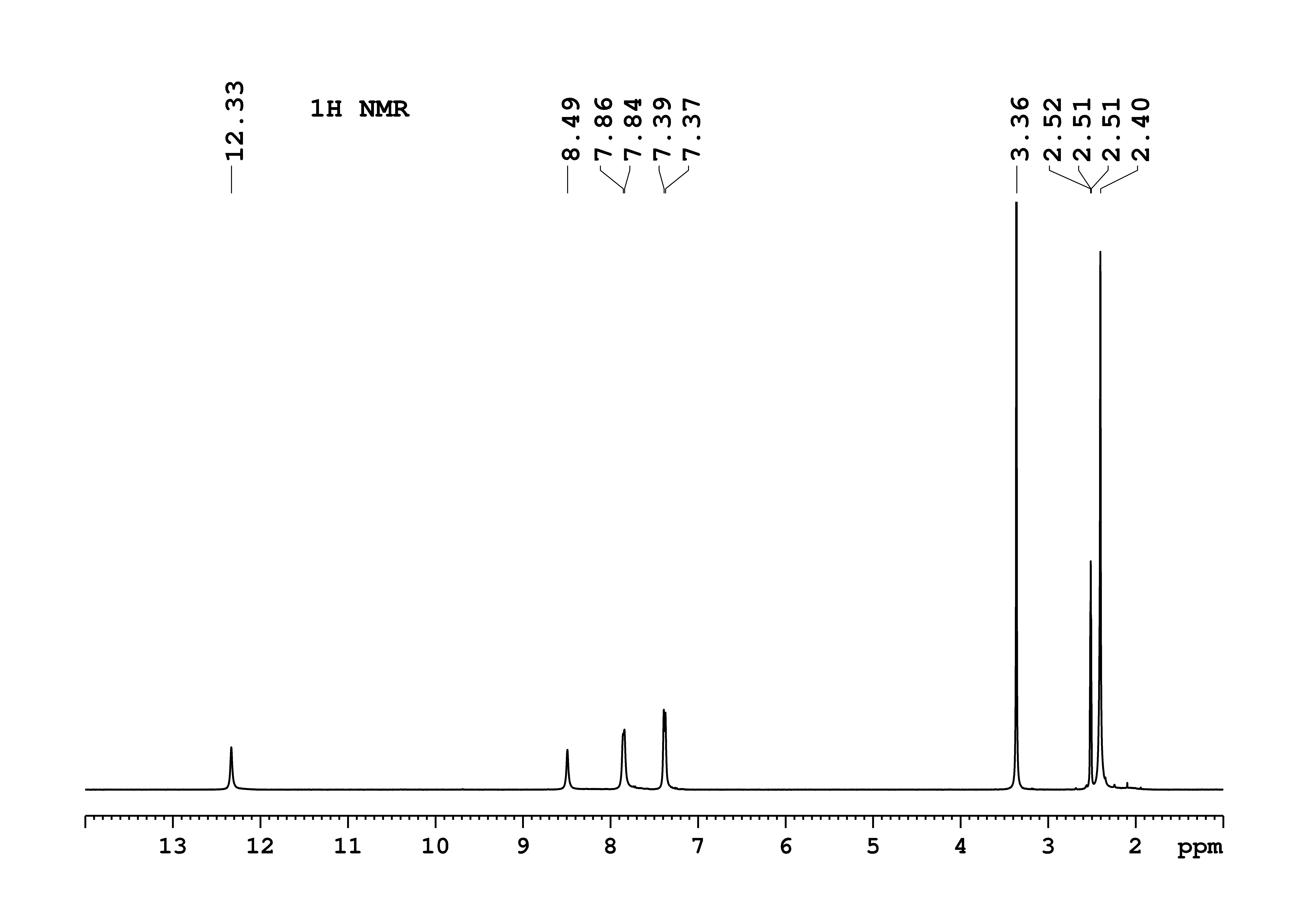


**Figure S10** ^1^H-NMR for Compound **5** in DMSO-d6.

**Figure S11** ^15^N-NMR for Compound **5** in DMSO-d6.


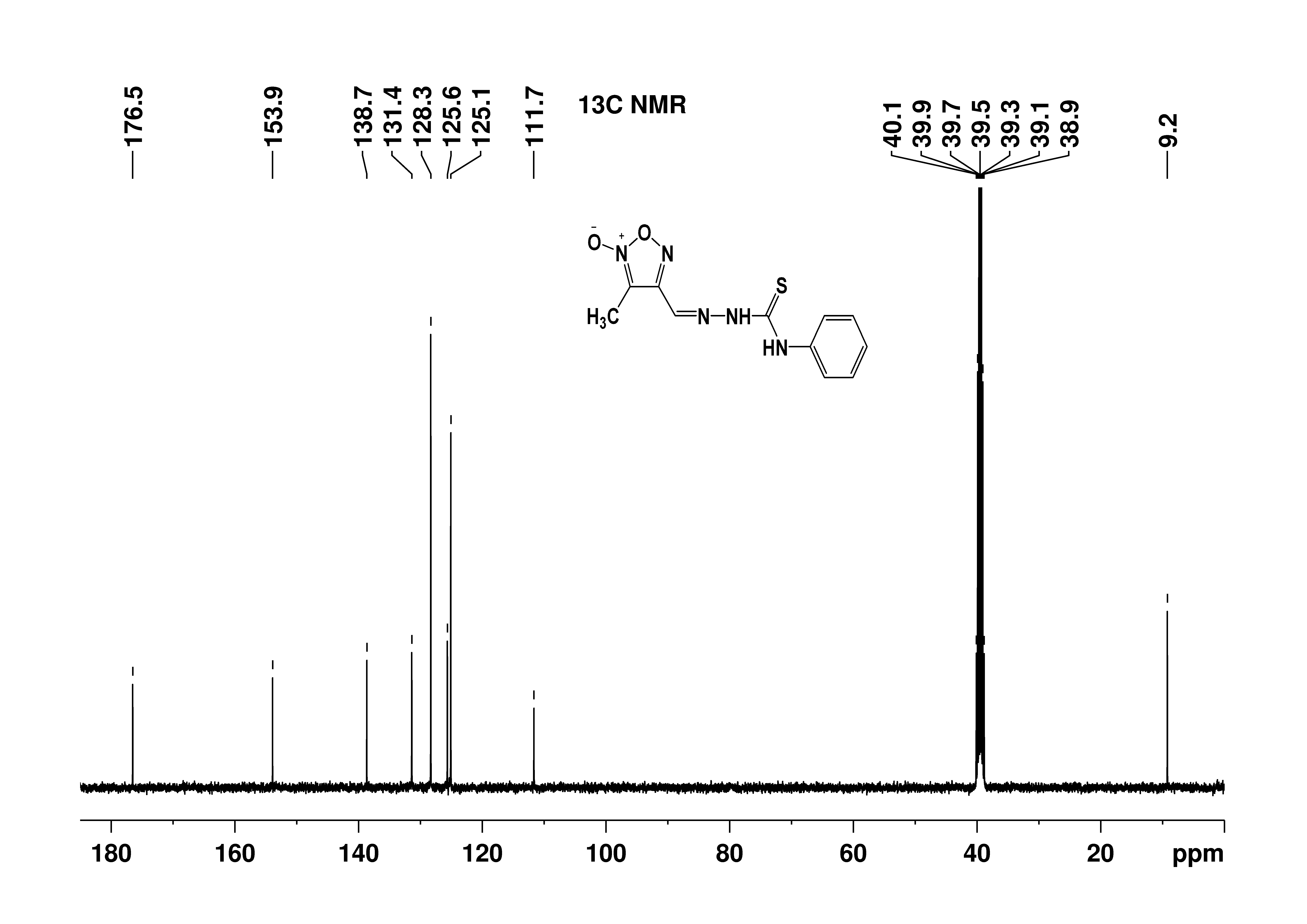


**Figure S12** ^13^C-NMR for Compound **7a** in DMSO-d6.


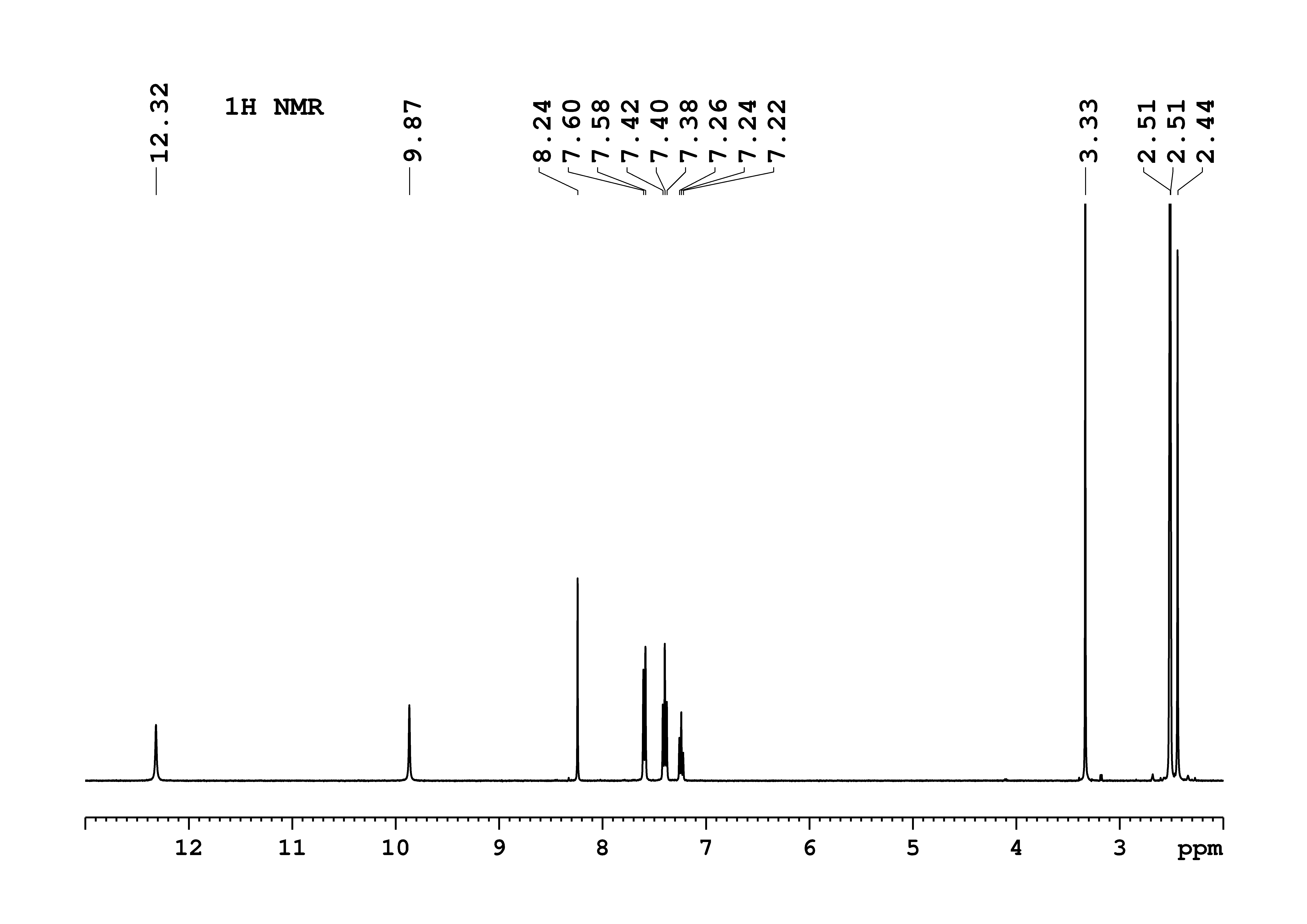


**Figure S13** ^1^H-NMR for Compound **7a** in DMSO-d6.

**Figure S14** ^15^N-NMR for Compound **7a** in DMSO-d6.


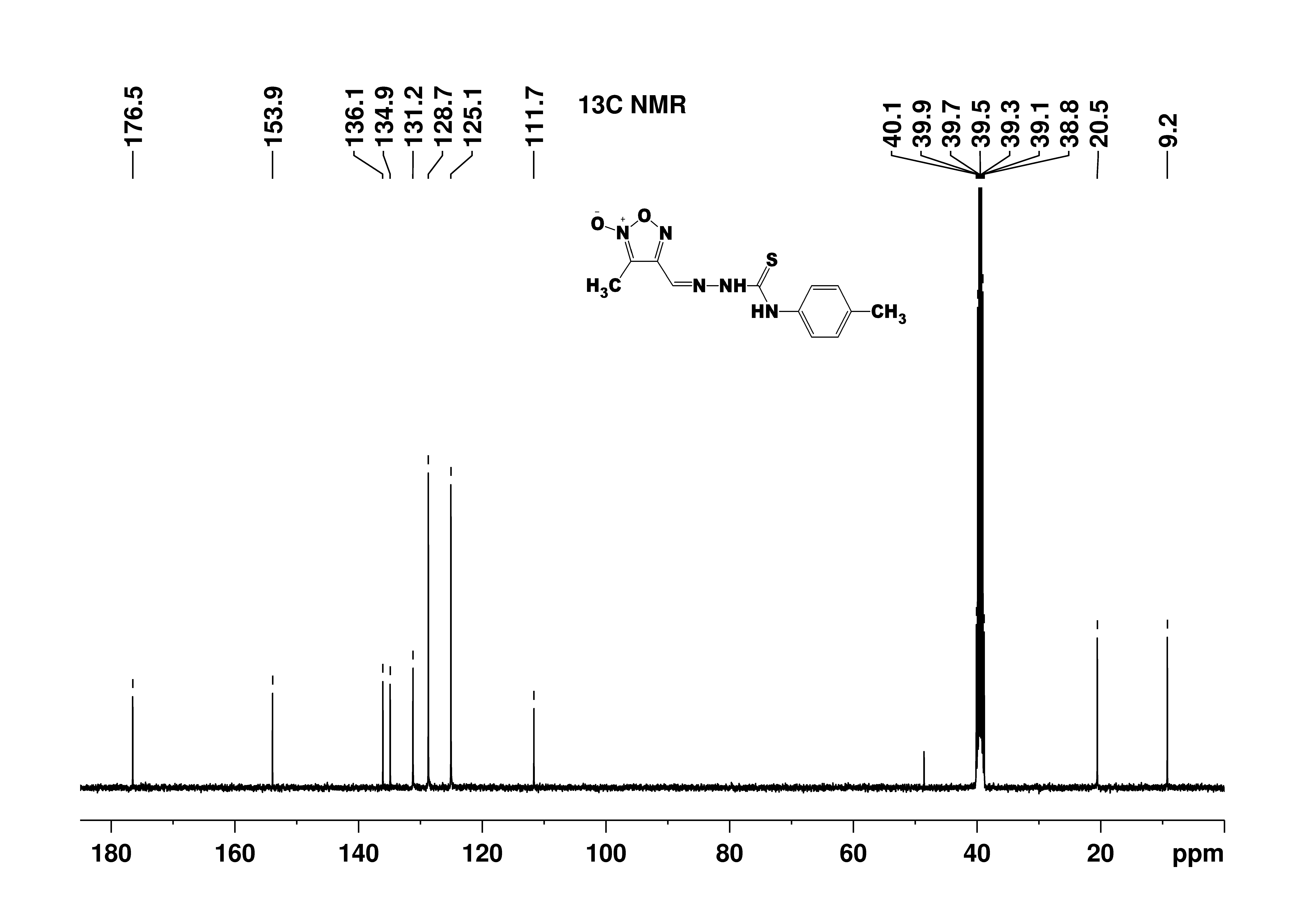


**Figure S15** ^13^C-NMR for Compound **7b** in DMSO-d6.


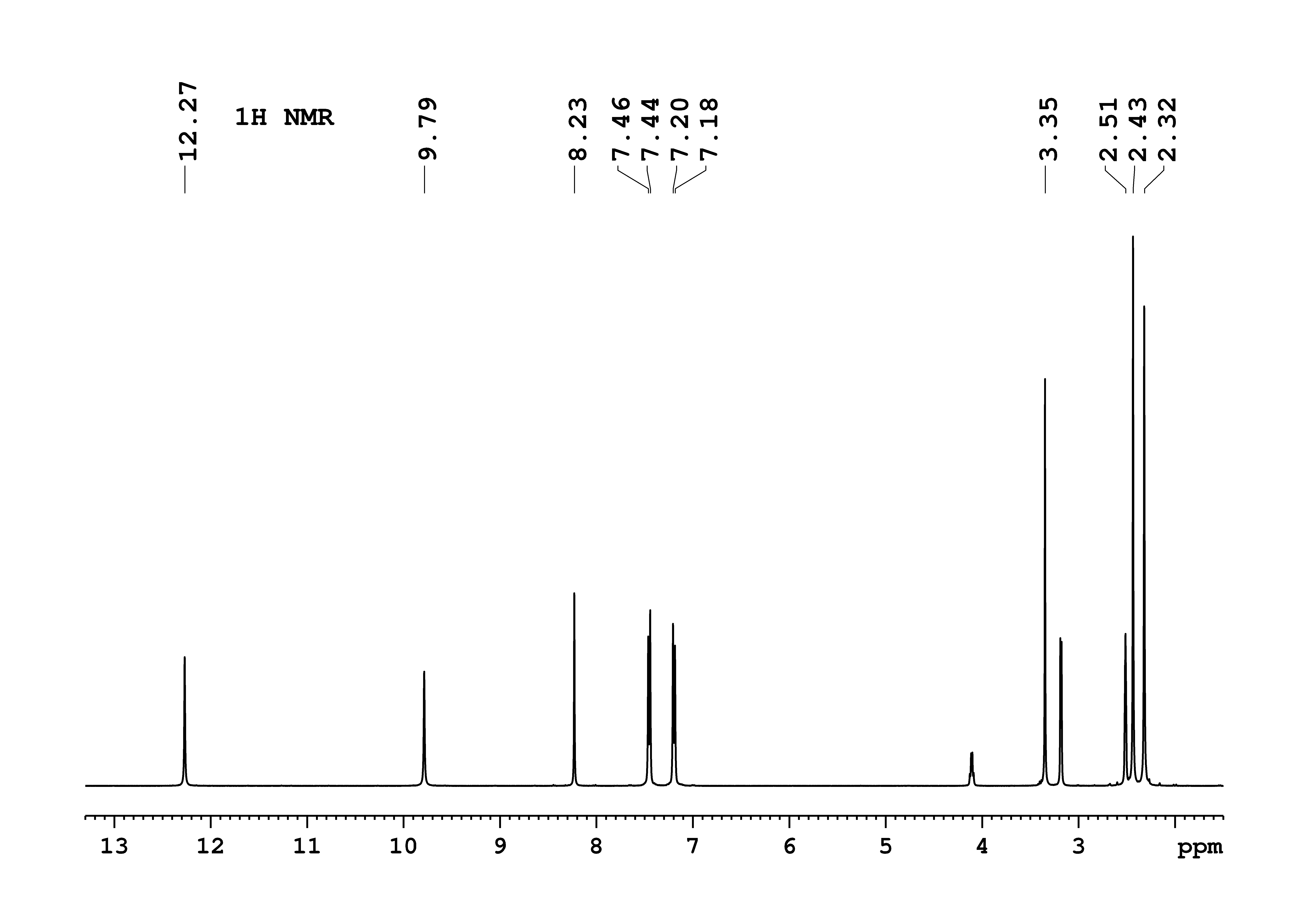


**Figure S16** ^1^H-NMR for Compound **7b** in DMSO-d6.

**Figure S17** ^15^N-NMR for Compound **7b** in DMSO-d6.
